# Development of a Performance-Based Measure of Executive Functions in Patients with Schizophrenia

**DOI:** 10.1371/journal.pone.0142790

**Published:** 2015-11-12

**Authors:** En-Chi Chiu, Shu-Chun Lee, Chian-Jue Kuo, For-Wey Lung, I-Ping Hsueh, Ching-Lin Hsieh

**Affiliations:** 1 Institute of Epidemiology and Preventive Medicine, College of Public Health, National Taiwan University, Taipei, Taiwan; 2 Taipei City Psychiatric Center, Taipei City Hospital, Taipei, Taiwan; 3 School of Occupational Therapy, College of Medicine, National Taiwan University, Taipei, Taiwan; 4 Department of Psychiatry, School of Medicine, Taipei Medical University, Taipei, Taiwan; 5 Psychiatric Research Center, Taipei Medical University Hospital, Taipei, Taiwan; 6 Department of Physical Medicine and Rehabilitation, National Taiwan University Hospital, Taipei, Taiwan; West China Hospital of Sichuan University, CHINA

## Abstract

A performance-based measure for assessing executive functions (EF) is useful to understand patients’ real life performance of EF. This study aimed to develop a performance-based measure of executive functions (PEF) based on the Lezak model and to examine psychometric properties (i.e., unidimensionality and reliability) of the PEF using Rasch analysis in patients with schizophrenia. We developed the PEF in three phases: (1) designing the preliminary version of PEF; (2) consultation with experts, cognitive interviews with patients, and pilot tests on patients to revise the preliminary PEF; (3) establishment of the final version of the PEF and examination of unidimensionality and Rasch reliability. Two hundred patients were assessed using the revised PEF. After deleting items which did not satisfy the Rasch model’s expectations, the final version of the PEF contained 1 practice item and 13 test items for assessing the four domains of EF (i.e., volition, planning, purposive action, and effective performance). For unidimensional and multidimensional Rasch analyses, the 4 domains showed good reliability (i.e., 0.77–0.85 and 0.87–0.90, respectively). Our results showed that the PEF had satisfactory unidimensionality and Rasch reliability. Therefore, clinicians and researchers could use the PEF to assess the four domains of EF in patients with schizophrenia.

## Introduction

Executive dysfunction is a major cognitive impairment in patients with schizophrenia [1,2]. Executive dysfunctions affect the ability of patients with schizophrenia performing activities of daily living (ADL), especially instrumental ADL (IADL) [3,4]. Patients who cannot perform ADL independently become a burden to their caregivers. Improvement of executive functions (EF) is one of the important treatment goals in patients with schizophrenia. Therefore, assessing EF in patients with schizophrenia is critical for clinicians and researchers to identify patients’ EF status and develop treatment plans.

Performance-based measures have been used to assess EF in patients with schizophrenia, such as the Executive Function Performance Test, the Multiple Errands Test, and the Virtual Action Planning Supermarket. Examiners ask examinees to perform functional activities (e.g., IADL) and observe their performance. The test results provide information on real life performance of EF [5], which is important for clinicians and researchers in order to understand patients’ actual behavior of EF in community living [6]. However, these three measures were not developed based on an EF theoretical framework, which may limit explanations of the construct being measured. Moreover, test items were not developed for patients with schizophrenia. The test items may be too difficult, so patients may give up and not want to perform them. Therefore, to resolve the abovementioned flaws, developing a new performance-based measure is necessary to assess EF in patients with schizophrenia.

The Lezak model is one of the widely used theoretical EF frameworks [7]. The Lezak model conceptualizes EF as a multidimensional construct, including four domains (i.e., volition, planning, purposive action, and effective performance) [8]. Volition domain refers to the ability to generate goals. Planning domain refers to the ability to identify and organize the steps or materials for achieving a goal. Purposive action domain refers to the ability to initiate, maintain, switch, and stop sequences of planned actions. Effective performance domain refers to the ability to monitor and correct mistakes. According to the definitions of the four domains, clinicians and researchers can understand how patients’ EF status influences their daily performance [9]. Therefore, the Lezak model can be considered for developing a performance-based measure assessing EF.

The purpose of this study was to develop a performance-based measure of executive functions (PEF) on the basis of the Lezak model to assess EF in patients with schizophrenia. We used IADL items that patients with schizophrenia have difficulty performing to create PEF items. We also examined psychometric properties (i.e., unidimensionality and reliability) of each domain in the PEF using Rasch analysis for patients with schizophrenia. Unidimensionality is a type of construct validity for determining whether the items of a domain reflect a single underlying construct [10]. Rasch reliability refers to the degree to which the measure is free from measurement error (i.e., degree of measurement precision) [11].

## Methods

We developed the PEF in three phases: (1) development of the preliminary PEF; (2) revision of the preliminary PEF; and (3) establishment of the final version of the PEF and examination of unidimensionality and Rasch reliability. The protocol of this study was approved by the Institutional Review Board of Taipei City Hospital. All participants were able to make voluntary and informed decisions and signed their written informed consent to participate in this study. Outpatients with schizophrenia were recruited from one psychiatric medical teaching hospital in northern Taiwan.

### Phase 1: development of the preliminary PEF

#### Step 1: exploring IADL items that patients with schizophrenia have difficulty performing

We reviewed published original articles using IADL measures to assess patients with schizophrenia within January 2008-May 2013. We compiled IADL items from the IADL measures and added IADL items according to suggestions from experts. This IADL questionnaire was used to interview patients with schizophrenia, caregivers, and psychiatric clinicians to investigate whether patients with schizophrenia have difficulty performing these IADL items. The inclusion criteria for the patients were: (1) diagnosis of schizophrenia based on the Diagnostic and Statistical Manual of Mental Disorders, fourth edition, text revision (DSM-IV-TR); (2) aged over 20 years; (3) diagnosed as schizophrenia for > 2 years; (4) no obvious general cognitive impairment with a score of ≥ 24 on the Mini Mental State Examination (MMSE); and (5) stable and consistent dose of antipsychotic medication received for three months. The exclusion criteria for the patients were: (1) history of severe brain injury; and (2) diagnoses of substance abuse. The inclusion criterion for the caregivers was main caregiver and living with the patient for > 5 years. The inclusion criterion for the clinicians was clinical experience in taking care of patients with schizophrenia for > 5 years.

The MMSE was used to represent participants’ global cognitive function. The total score of the MMSE ranges from 0 to 30. Higher scores demonstrate better global cognitive function [12]. The MMSE has acceptable test-retest reliability in patients with schizophrenia [13,14].

#### Step 2: designing the preliminary PEF using IADL items

After conducting interviews, we chose IADL items for designing the preliminary PEF with the following 4 standards: (1) items having minimal gender and cultural differences; (2) items that could be performed with minimal danger (e.g., using a kitchen knife was not chosen); (3) items that could be implemented in a room; and (4) items that > 25% of all interviewees indicated patients with schizophrenia having difficulty performing. We chose the item which the lowest percentage of interviewees indicated that patients with schizophrenia had difficulty performing to be the “practice item”. For each chosen IADL item, we designed the instructions, scoring criteria and testing materials to assess the four domains based on the Lezak model.

### Phase 2: revision of the preliminary PEF

#### Step 1: consultation with experts to revise the preliminary PEF

We conducted expert review to revise the preliminary PEF. Experts who did not participate in Phase 1 received the preliminary PEF via e-mail, and gave comments for item appropriateness, description of instructions, materials used, and scoring criteria of the preliminary PEF. In each round of consultation, we compiled experts’ comments, revised the preliminary PEF, and asked for experts’ agreement or disagreement of the revision. The consultation was repeated until at least 80% of the experts agreed with the revision (named as PEF draft-1).

#### Step 2: cognitive interviews of the PEF draft-1

Cognitive interviews of the PEF draft-1 were conducted with patients to confirm the clarity of the item instructions. The patients were recruited with the same criteria as Step 1 of Phase 1. Proceeding item by item, the first author tested and interviewed each patient using three open-ended questions: (1) In your words, what would you think this instruction was asking? (2) Was this instruction easy to understand? Were there any specific words that are difficult to understand? (3) How would you change the words to make them easier to understand? After each cognitive interview, we compiled the comments and revised the instructions. The process of testing and revision was repeated until no more comments were given. The PEF draft-2 was completed at this stage.

#### Step 3: pilot tests of the PEF draft-2

We conducted pilot tests on small groups of patients to examine the appropriateness of the administrative procedures and scoring criteria. The inclusion and exclusion criteria of recruited patients were the same as Step 1 of Phase 1, except for MMSE ≥ 24. We stopped the pilot testing when the data met the four rules: (1) Cronbach’s alpha (α) ≥ 0.90 in each domain of the PEF draft-2; (2) corrected item-total correlation ≥ 0.30 for each item of each domain [15]; (3) percentage of patients rated on each response category in the range of 10%-75% [16–18]; and (4) no ceiling effect and floor effect for a domain (< 20% of the total score in maximum and minimum scores, respectively) [19]. The PEF draft-3 was completed at this stage.

### Phase 3: establishment of the final version of the PEF and examination of unidimensionality and Rasch reliability

Patients with schizophrenia were recruited between December 2013 and February 2014. The patients were recruited with the same criteria as Step 3 of Phase 2. The patients’ demographic data were collected from medical records.

We examined the unidimensionality of each domain in the PEF draft-3 using Rasch rating scale model in the WINSTEPS computer program. This computer program has been used to conduct Rasch analysis in research for patients with schizophrenia [20–22]. To examine unidimensionality, we first used infit and outfit statistics to evaluate whether the data fit the model’s expectations. Items with infit or outfit mean square error (MNSQ) < 0.6 or > 1.4 indicated misfit [23]. The misfit item was deleted in the four domains and then we reconducted Rasch analysis until the items fitted the criteria of infit and outfit MNSQ simultaneously in the four domains (the final version of the PEF). Second, we confirmed unidimensionality using principal component analysis (PCA) on the standardized residuals. We estimated the eigenvalue of the residual variance (unexplained variance) in the first contrast. The criterion of unidimensionality was the eigenvalue of the first contrast < 2.0 (i.e., the strength of the first contrast is less than 2 items, which is not able to form a dimension) [24,25].

We calculated Rasch reliability of the four domains in the final version of the PEF using unidimensional and multidimensional Rasch analyses. The criterion of the reliability coefficient was ≥ 0.70 [26]. We performed the multidimensional analysis using ConQuest software.

## Results

### Phase 1

**Fig 1:**
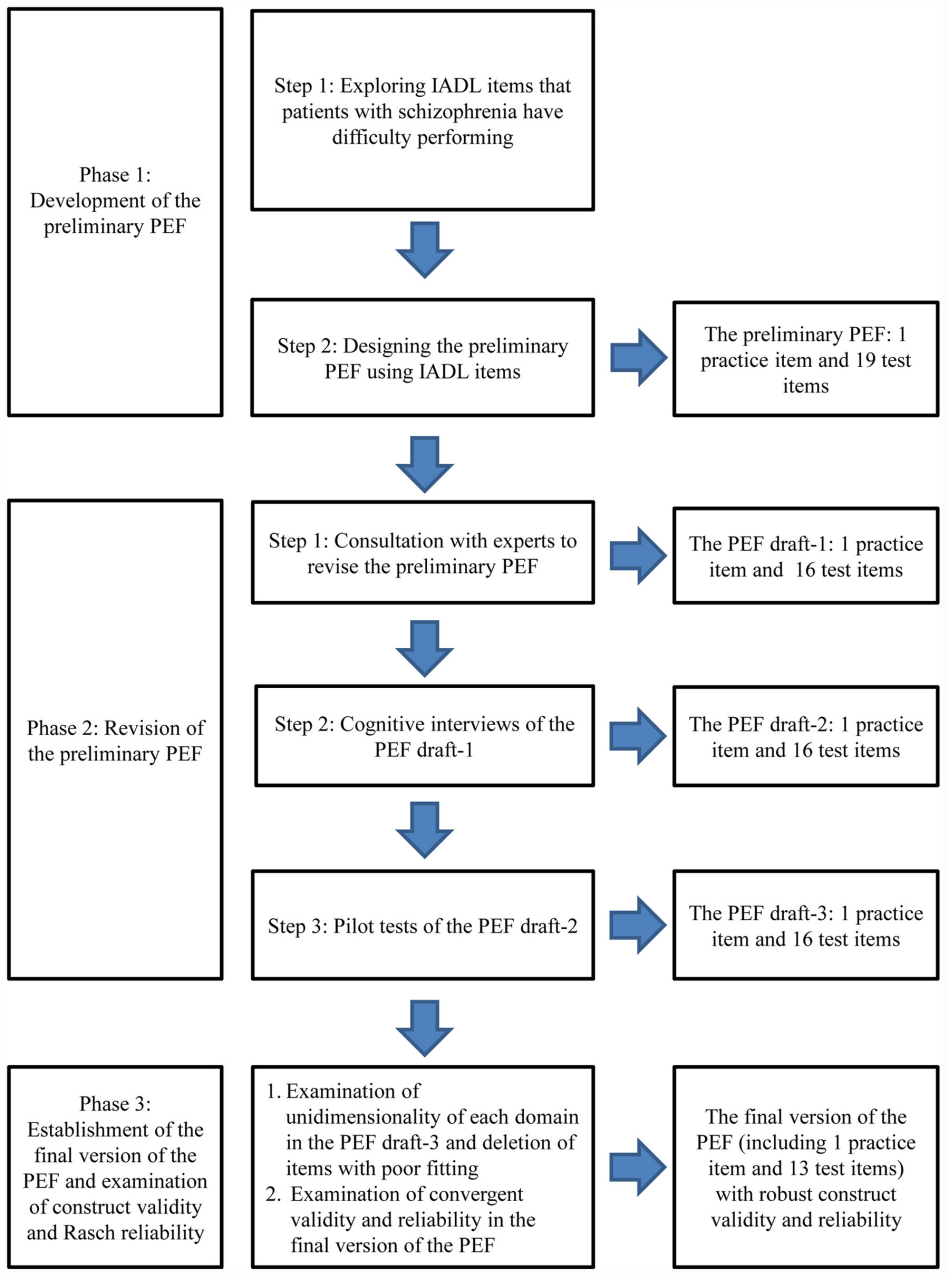
The flowchart of developing the PEF.

Fig 1 presents the process and results of the three phases in developing the PEF.

#### Step 1

From the literature review, we complied 41 IADL items from the 19 IADL measures. Four experts (i.e., 2 psychiatric occupational therapists and 2 researchers in the field of IADL) suggested 6 additional IADL items. Thus, the IADL questionnaire included 47 items. We used the IADL questionnaire to conduct interviews with 32 patients, 27 caregivers, and 12 psychiatric clinicians.

#### Step 2

According to the preset 4 standards, we chose 19 IADL items for designing the preliminary PEF. The 19 IADL items could be divided into 7 categories as follows: (1) communication management: using fax machine, addressing envelope, and sending e-mail; (2) community mobility: using street map and using bus route map; (3) financial management: paying bill, withdrawing money from an ATM machine, filling out deposit slip, filling out withdrawal slip, and shopping under budget; (4) health management: medicine management and diet control; (5) home management: hanging out clothes, folding clothes, using washing machine, and sorting garbage; (6) cooking: using microwave and using electric stove; (7) shopping: buying necessities. The practice item was “using telephone”.

For each IADL item, we designed three instructions. The first instruction was to ask what a patient would do for a task in a given context for assessing the volition domain. In the volition domain, the examiner rated whether examinees could set an appropriate goal. The second instruction was to ask how the patient would execute the task for assessing the planning domain. In the planning domain, the examiner rated whether examinees could identify and organize the steps or materials for reaching a goal. The third instruction was to ask the patient to physically perform the task for assessing the other two domains (i.e., purposive action and effective performance). In the purposive action domain, the examiner rated whether examinees performed the sequence of actions for a task. In the effective performance domain, the examiner rated whether examinees monitored and corrected mistakes during the task.

### Phase 2

#### Step 1

We invited 10 experts to participate, including 2 psychiatrists, 4 psychiatric occupational therapists, and 4 researchers in the fields of cognition and psychiatry. According to the experts’ comments, we deleted two items (i.e., using fax machine and filling out withdrawal slip), because the content of these items were similar to using telephone and filling out deposit slip, respectively. We considered age difference, and deleted one item (sending e-mail), because elderly people may not know how to send e-mails. In this stage, the PEF draft-1 contained 1 practice item and 16 test items, which included 51 instructions.

#### Step 2

Three rounds of cognitive interviews were conducted for the 51 instructions. We recruited ≥ 8 patients in each round. In rounds 1 and 2, patients could understand 86% (44/51) and 94% (48/51) of the instructions, respectively. We revised the ambiguous or difficult wordings of instructions based on the patients’ comments. In round 3, no comments on the instructions (named as PEF draft-2) were reported from the patients. The patients in this step overlapped with those who received the IADL questionnaire interview in Step 1 of Phase 1.

#### Step 3

Five pilot tests were conducted. We recruited ≥ 10 patients in each pilot test. In the first four pilot tests, the results did not meet the four rules that were used to stop the pilot testing. According to the results of the first four rounds, we modified the number of instructions given, item order, and scoring criteria in the PEF draft-2. In the fifth pilot test, the four rules were met. For the four domains of the PEF draft-3, Cronbach’s α was ≥ 0.91 and corrected item-total correlation coefficient was ≥ 0.40. The percentage of patients rated on each response category was 15%-70%. No obvious ceiling and floor effects were observed (≤ 10%).

### Phase 3

A convenience sample of 200 patients with schizophrenia was recruited, including test data from the fifth pilot test. Their mean age was 43.5 years, and 45.0% of patients were male. The mean score of the MMSE was 25.2. Further details of the patients are shown in Table 1.

**Table 1:** Characteristics the patients with schizophrenia (n = 200).

Characteristic	
Gender n (%)	
Male	90 (45.0)
Female	110 (55.0)
Age (mean year [SD])	43.5 (10.5)
Onset age (mean year [SD])	22.1 (7.4)
Duration of illness (mean year [SD])	21.8 (9.5)
Education n (%)	
Elementary school	9 (4.5)
Junior high school	17 (8.5)
Senior high school	99 (49.5)
College and above	75 (37.5)
Schizophrenia subtypes n (%)	
Simple type	43 (21.5)
Disorganized type	6 (3.0)
Paranoid type	33 (16.5)
Schizophreniform disorder	2 (1.0)
Residual type	3 (1.5)
Schizoaffective disorder	7 (3.5)
Undifferentiated type	106 (53.0)
Type of antipsychotics n (%)	
First generation	67 (33.5)
Second generation	161 (80.5)
Third generation	5 (2.5)
Taking two types of antipsychotics	33(16.5)
Mini Mental State Examination (mean [SD])	25.2 (4.3)

**Fig 2:**
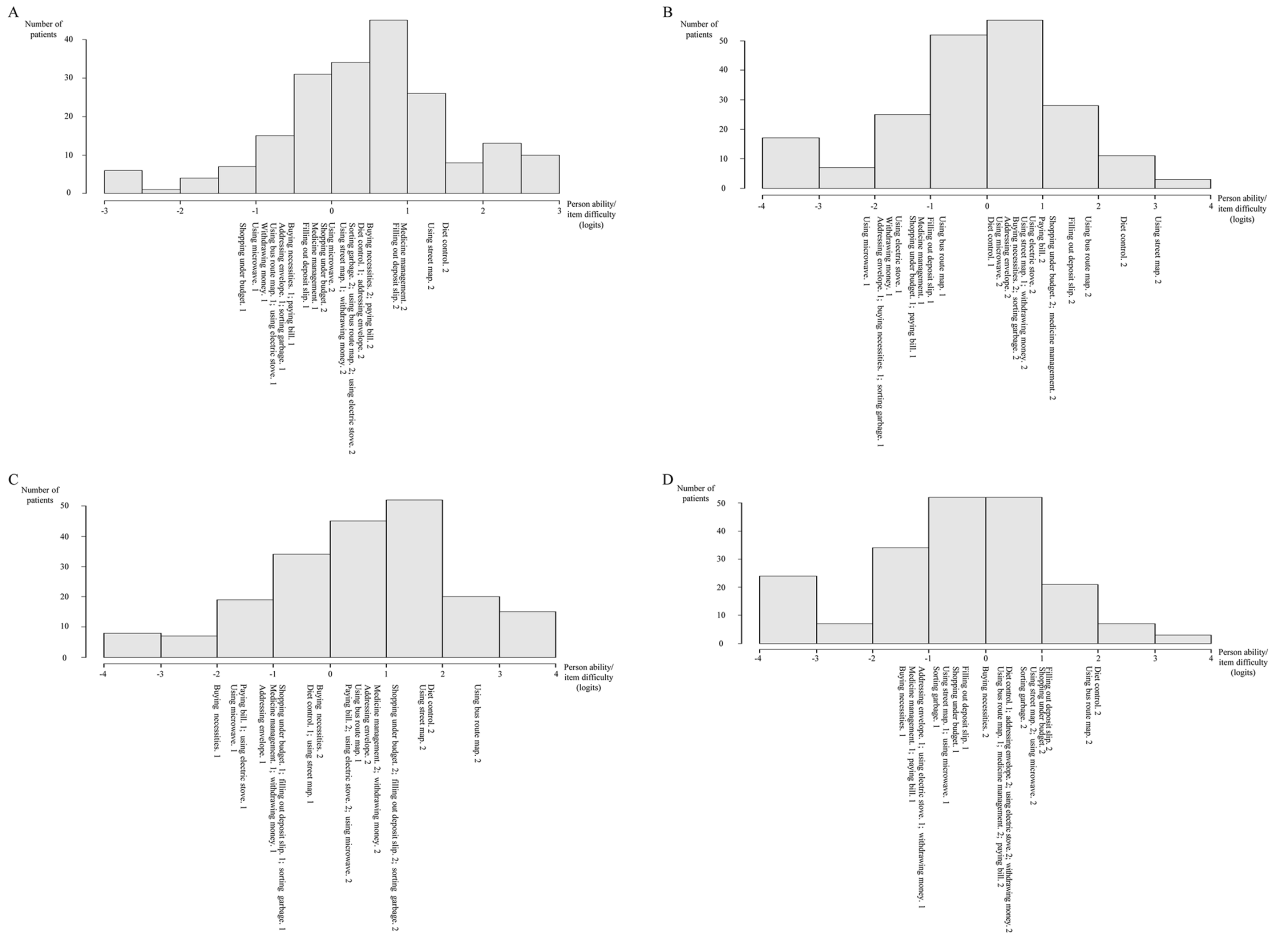
Item-person map. (A) volition; (B) planning; (C) purposive action; and (D) effective performance. The numbers after the decimal point for each item indicate the step difficulty. In a 3-point scale (0–2), for example, “diet control. 1” represents the first step difficulty (between response categories 0 and 1) of the “diet control” item and “diet control. 2” represents the second step difficulty (between response categories 1 and 2).

For examining unidimensionality, three items were deleted (i.e., hanging out clothes, folding clothes, and using washing machine), because the outfit MNSQs were higher than the preset criterion (1.4) in one or two domains. The outfit MNSQ of the “folding clothes” item was 1.57 in the volition domain. The outfit MNSQ of the “hanging out clothes” item was 1.50 in the effective performance domain. The outfit MNSQs of the “using washing machine” item were 1.42 and 1.49 in the purposive action and effective performance domains, respectively. Table 2 shows estimates of difficulty, infit MNSQ, and outfit MNSQ for the 13 remaining test items. Fig 2 (item-person map) presents the order of item difficulty corresponding with the distribution of person ability. Regarding the residuals in PCA for these 13 items, the eigenvalue of the first contrast was 1.5–1.7 in the 4 domains (Table 3). The final version of the PEF contained 1 practice item (using telephone) and 13 test items as follows: (1) communication management: addressing envelope; (2) community mobility: using street map and using bus route map; (3) financial management: paying bill, withdrawing money from an ATM machine, filling out deposit slip, and shopping under budget; (4) health management: medicine management and diet control; (5) home management: sorting garbage; (6) cooking: using microwave and using electric stove; (7) shopping: buying necessities (Appendix in S1 File). The items of each domain had three response categories ranging from 0 to 2. The general scoring criteria for the volition and planning domains were: 0, no response or response not related to context; 1, response related to part of context; and 2, response related to complete context. The scoring criteria of the purposive action domain were: 0, doing ≤ 1 necessary step of the task; 1, doing ≥ 2 necessary steps of the task, but not completing the task; 2, doing all necessary steps of the task. The scoring criteria of the effective performance domain were: 0, making ≥ 2 mistakes; 1, making 1 mistake; 2, not making any mistakes.

**Table 2:** Estimates of difficulty, infit MNSQ, and outfit MNSQ in the final version of PEF.

	Volition			Planning			Purposive action			Effective performance		
Item	Difficulty logit^a^	Infit MNSQ	Outfit MNSQ	Difficulty logit^a^	Infit MNSQ	Outfit MNSQ	Difficulty logit^a^	Infit MNSQ	Outfit MNSQ	Difficulty logit ^a^	Infit MNSQ	Outfit MNSQ
1. Sorting garbage	-0.21	0.96	0.90	-0.64	0.99	0.92	0.18	1.07	1.08	-0.09	1.03	1.14
2. Filling out deposit slip	0.29	1.16	1.07	0.21	0.80	0.87	0.16	0.95	0.96	0.30	1.02	0.93
3. Buying necessities	-0.03	0.89	0.90	-0.59	1.12	1.07	-1.05	0.88	0.82	-0.78	0.99	0.98
4. Using electric stove	-0.22	0.89	0.99	-0.36	0.82	0.83	-0.64	0.95	1.05	-0.36	0.99	1.16
5. Diet control	0.96	0.93	0.86	1.28	1.10	1.21	0.71	1.04	1.14	1.17	0.95	1.23
6. Withdrawing money	-0.32	0.88	0.80	-0.47	0.90	0.89	-0.09	1.28	1.13	-0.30	1.18	1.07
7. Shopping under budget	-0.65	1.31	1.20	-0.06	0.97	0.90	0.12	1.12	1.08	0.20	1.06	1.04
8. Using microwave	-0.49	1.06	1.03	-0.91	1.04	1.00	-0.71	0.97	1.31	0.11	1.13	1.26
9. Medicine management	0.33	1.03	1.09	0.00	0.95	1.01	-0.08	0.85	1.09	-0.53	0.92	1.09
10. Using bus route map	-0.23	1.00	0.96	0.48	1.11	1.09	1.60	0.82	0.85	1.03	0.80	0.71
11. Paying bill	-0.05	1.12	0.93	-0.16	1.01	1.29	-0.62	1.24	0.94	-0.49	0.86	0.79
12. Using street map	0.75	0.82	1.00	1.94	0.99	1.03	0.62	0.91	0.84	0.04	0.98	0.88
13. Addressing envelope	-0.13	1.06	1.04	-0.72	1.16	1.10	-0.19	0.85	0.78	-0.30	1.00	0.93

^a^Mean value of two thresholds.

**Table 3:** Eigenvalue of the first contrast^a^ in the standardized residuals and reliability of the PEF.

Domain	Eigenvalue of the first contrast	Reliability (unidimensional Rasch analysis)	Reliability (multidimensional Rasch analysis)
Volition	1.5	0.77	0.87
Planning	1.5	0.85	0.88
Purposive action	1.6	0.85	0.90
Effective performance	1.7	0.83	0.90

^a^First contrast represents the size of the first component in the residuals.

For unidimensional Rasch analysis, reliability was 0.77–0.85 (Table 3) in the 4 domains of the PEF. For the multidimensional Rasch analysis, reliability was 0.87–0.90 in the 4 domains.

## Discussion

The purpose of this study was to develop the PEF for assessing EF in patients with schizophrenia. Using Rasch analysis, results from infit and outfit statistics, and PCA on the standardized residuals revealed that the 13 test items of the individual domains had unidimensionality. The results support that each domain assessed a single construct and the items’ scores of each domain could be summed up to represent patients’ level of functions. A higher score on each domain indicated better domain-specific function.

Rasch reliabilities in unidimensional or multidimensional Rasch analyses were good (coefficients≥0.77). The reliability of the domains using multidimensional Rasch analysis was higher (coefficients 0.87–0.90) than those using unidimensional Rasch analysis (coefficients 0.77–0.85). The reason is that correlations between domains were considered using the multidimensional approach, which can improve reliability of measurement [27]. The improvement of reliability in multidimensional Rasch analyses indicates that the items yield more precise estimates of patients’ abilities. Thus, we suggest using the multidimensional Rasch scores to represent patients’ EF functions. As a whole, the PEF has sufficient reliability to assess the four domains of EF in patients with schizophrenia.

We used the IADL items which patients with schizophrenia have difficulty performing to design the PEF. EF are higher-level cognitive functions, which are involved in performing non-routine activities in the non-automatic manner [6]. The IADL items were not done routinely and patients may not do the difficult IADL items automatically. Therefore, the difficult IADL items are considered for use to examine EF in this study.

The PEF contains three advantages for use in clinical and research settings. First, the PEF was developed based on the theoretical model (i.e., Lezak model), which can assess EF in a comprehensive manner (i.e., 4 domains). Second, the PEF contains a variety of IADL items (i.e., 7 categories), which can assess patients’ EF status in different aspects of daily life. Third, we used Rasch analysis to transform the four domains’ scores from ordinal to interval. Future users can apply Rasch interval scores to compare the PEF outcomes within an individual patient and between patients. Therefore, the PEF can be a useful tool to identify patients’ EF status in four different domains.

In the item-person map, our results showed that the 13 items covered the majority of patients in the four domains, indicating these items could differentiate the patients well. However, we observed obvious gaps between “using street map” and “medicine management” in the volition domain, between “diet control” and “using bus route map” in the planning domain, between “using bus route map” and “diet control” in the purposive action domain, and between” using bus route map” and “filling out deposit slip” in the effective performance domain. Adding new items could increase the spread of item difficulties to cover different levels of patients’ functions. However, administering the 13-item PEF takes about 40 minutes, which may be time-consuming. Future studies could develop a computerized adaptive test with more items to assess patients in a more efficient manner.

Three items showed poor-fitting in outfit MNSQs (> 1.4) in volition, purposive action, and/or effective performance domains, which indicates that patients may respond to items with lucky guesses and carelessness [10]. Instruction 1 of the “folding clothes” item was “If the clothes are taken off from the hanger and put in the closet, what would you do?” Patients who are used to hanging up clothes in the closet may be confused by the situation (why do the clothes need to be taken off from the hanger before putting them in the closet) and reply by guessing. To reduce confusion, Instruction 1 of this item may be changed to “If dry clothes are taken off from the hanger, what would you do?” For the “hanging out clothes” item, Instruction 3 was that patients were asked to hang out two pieces of clothing (i.e., t-shirt and pants) and turn them to the correct side. Patients may make a mistake (e.g., turn only one piece of clothing to the correct side), because of carelessness. The scoring criteria of the effective performance domain may consider not counting “turn only one piece of clothing to the correct side” to be a mistake and accept this as a correct response. Instruction 3 of the “using washing machine” item was that patients were asked to operate the buttons on the washing machine. The washing machine did not give feedback and patients may fiddle with the buttons. Developing a virtual measure which provides instant feedback could reduce fiddling with the buttons on the washing machine. Future studies may revise the deleted items according to our suggestions.

This study has three limitations. First, this study used a convenience sample, which may limit the generalization of our findings. Future studies with the same schizophrenic populations are needed to cross-validate our findings. Second, we used the Rasch model (i.e., one-parameter), which did not consider item discrimination. Further studies could use the item response model with more parameters to further validate our findings. Third, intra-rater and inter-rater reliabilities of the PEF have not been examined, which may restrict the explanation of test results. Future studies are needed to examine intra-rater and inter-rater reliabilities of the PEF.

In summary, unidimensionality and Rasch reliability of the PEF were supported. The PEF has the potential to be used for assessing EF in patients with schizophrenia in both clinical and research settings.

## Supporting Information

S1 File Appendix. Final version of the PEF.
(DOCX)
