# Supplementary material for: Development of a Performance-Based Measure of Executive Functions in Patients with Schizophrenia
Source: PLoS One. 2015 Nov 12;10(11):e0142790. doi: 10.1371/journal.pone.0142790 (PMC4642955; doi:10.1371/journal.pone.0142790)
Supplement: S1 File — Final version of the PEF. (DOCX) [file pone.0142790.s001.docx]

| Practice item: using telephone  Instruction 1: If you need to order boxed meals and have them delivered, what would you do?  Instruction 2: Have you ever made a phone call to order boxed meals? If you wanted to make a phone call to order 10 pork chop boxed meals, how would you do it? Please describe the steps as detailed as possible.  Instruction 3: Now you have to do a task. Please dial the phone number of the boxed meal shop and pretend you want to order 10 pork chop boxed meals and have them sent to this address. After making this call, please give me the telephone list. You can start when I say “go”. Do it as quickly as possible. Go! |
| --- |
| 1. Sorting garbage  Instruction 1: If you want to be environmentally friendly and you have a pile of garbage, what would you do?  Instruction 2: Have you ever sorted garbage? If you have a pile of garbage and need to sort them, how would you do it? Please describe the steps as detailed as possible.  Instruction 3: Now you have to do a task. Please sort these garbage according to the garbage sorting sheet. After sorting, please give me the plastic bag. You can start when I say “go”. Do it as quickly as possible. Go! |
| 2. Filling out deposit slip  Instruction 1: If you plan to go overseas one year later and you don’t have enough money now, and you need to manage your earning, what would you do?  Instruction 2: Have you ever deposited money in the bank or post office? If you were in the bank or post office and ready to deposit money, how would you do it? Please describe the steps as detailed as possible.  Instruction 3: Now you have to do a task. Pretend you are in the bank and need to deposit NT$5000 for yourself, please fill out the necessary information on the deposit slip. After filling it out, put the money and deposit slip on the counter. You can start when I say “go”. Do it as quickly as possible. Go! |
| 3. Buying necessities  Instruction 1: If there is an annual sale in the supermarket and the necessities you needed are on sale, what would you do?  Instruction 2: Have you ever bought things in the supermarket? If you are in the supermarket and you need to buy things and go home, how would you do it? Please describe the steps as detailed as possible.  Instruction 3: Now you have to do a task. According to the shopping list, Please circle the things you need to buy on the paper (pictures of items). After circling them, put this paper on the shopping cart. You can start when I say “go”. Do it as quickly as possible. Go! |
| 4. Using electric stove  Instruction 1: If there is a hot pot and you need to heat the food, what would you do?  Instruction 2: Have you ever used an electric stove? If there is a hot pot and you need to heat the food using an electric stove, how would you do it? Please describe the steps as detailed as possible.  Instruction 3: Now you have to do a task. Pretend there is food in the pot and you need to heat the food using an electric stove and set the power level at 3. You can start when I say “go”. Do it as quickly as possible. Go! |
| 5. Diet control  Instruction 1: If you are on a diet and need to make sure that calories are not too high, what would you do?  Instruction 2: Have you ever calculated the calories? If you wanted to know the total calories for breakfast, how would you do it? Please describe the steps as detailed as possible.  Instruction 3: Now you have to do a task. Please tell me two kinds of food and one kind of drink for breakfast, but the total calories cannot exceed 500 calories. You can start when I say “go”. Do it as quickly as possible. Go! |
| 6. Withdrawing money  Instruction 1: If you have savings in the bank and needed to use the money in your account, but the bank was closed, what would you do?  Instruction 2: Have you ever withdrawn money from the ATM machine? If you need to withdraw money from the ATM machine, how would you do it? Please describe the steps as detailed as possible.  Instruction 3: Now you have to do a task. Pretend that you inserted a card into the ATM machine. Please withdraw NT$1000 from the ATM machine and choose not to print out the receipt. You can start when I say “go”. Do it as quickly as possible. Go! |
| 7. Buying under budget  Instruction 1: If you need to stay healthy and eat fruit everyday, but there is no fruit at home, what would you do?  Instruction 2: Have you ever bought fruit? If you need to buy fruit with only the money you have on you, how would you do it? Please describe the steps as detailed as possible.  Instruction 3: Now you have to do a task. Pretend you have this much money. Please buy two kinds of fruit and put them in the plastic bag. After you are finished, put the plastic bag and money on the counter. You can start when I say “go”. Do it as quickly as possible. Go! |
| 8. Using microwave  Instruction 1: If you need to eat a hot boxed meal and complete the heating in 2 minutes, what would you do?  Instruction 2: Have you ever used a microwave? If you needed to heat a boxed meal using a microwave, how would you do it? Please describe the steps as detailed as possible.  Instruction 3: Now you have to do a task. Pretend there is food in the boxed meal and the cover cannot be heated. Please heat this boxed meal for 2 minutes using the microwave. You can start when I say “go”. Do it as quickly as possible. Go! |
| 9. Medicine management  Instruction 1: If you see a doctor and get different medicines, and need to remind yourself to take medicine at a specific time, what would you do?  Instruction 2: Have you ever used a pill box? If you need to arrange medicines using a pill box, how would you do it? Please describe the steps as detailed as possible.  Instruction 3: Now you have to do a task. You need to prepare medicine for one week. Please arrange all the medicines in the pill box. After you are finished, give me the pill box. You can start when I say “go”. Do it as quickly as possible. Go! |
| 10. Using bus route map  Instruction 1: If a hospital is very far away from your friend’s house and you have to go your friend’s house after seeing the doctor. What would you do?  Instruction 2: Have you ever taken a bus by yourself? If you needed to take a bus from a hospital to your friend’s house, how would you do it? Please describe the steps as detailed as possible.  Instruction 3: Now you have to do a task. Pretend you need to go Xiaohua’s house from the hospital. Please tell me which bus route you should take and tell me which station you get on and which station you get off. You can start when I say “go”. Do it as quickly as possible. Go! |
| 11. Paying bill  Instruction 1: If you don’t want your home phone service to be suspended, what would you do?  Instruction 2: Have you ever paid the telephone fees by yourself? If you needed to pay the telephone fees now, what things would you need to take and how would you do it? Please describe the steps as detailed as possible.  Instruction 3: Now you have to do a task. There is money in the wallet. Please take out the exact money from the wallet to pay the telephone fees and then put the money and the bill on the counter. You can start when I say “go”. Do it as quickly as possible. Go! |
| 12. Using street map  Instruction 1: If you need to walk to a place where you have not been before and there isn’t anyone you could ask, what would you do?  Instruction 2: Have you ever used a map? If you need to use a map to walk from the gas station to the movie theater, how would you do it? Please describe the sequences as detailed as possible.  Instruction 3: Now you have to do a task. Pretend you are in the gas station now. You need to pass by three locations (library, movie theater, and market) and to meet your friend at the flower market in the end. Please draw the shortest route to pass through all the locations. You can start when I say “go”. Do it as quickly as possible. Go! |
| 13. Addressing envelope  Instruction 1: If you are holding a birthday gathering and need to give the invitation to a friend faraway, what would you do?  Instruction 2: Have you ever sent a letter? If you need to send a letter, what would you write on the envelope? Please describe the content as detailed as possible.  Instruction 3: Now you have to do a task. Pretend you need to send a letter to Xiao-Ming, Wang. Please fill out the necessary information on the envelope. After you are finished, put the envelope on the mailbox. You can start when I say “go”. Do it as quickly as possible. Go! |

Scoring criteria

| Domain | 0 | 1 | 2 |
| --- | --- | --- | --- |
| Volition | no response or response not related to context | response related to part of the context or not making an appropriate goal | response related to complete context and making an appropriate goal |
| Planning | no response or response not related to context | response related to part of the context | response related to complete context |
| Purposive action | no action or doing one necessary step of the task | doing ≥ 2 necessary steps of the task, but not completing the task | doing all necessary steps of the task and completing the task |
| Effective performance | no action or making ≥ 2 mistakes | making 1 mistake | not making any mistakes or making mistakes but correcting the mistakes |
